# Growth, Carcass Traits, Blood Hematology, Serum Metabolites, Immunity, and Oxidative Indices of Growing Rabbits Fed Diets Supplemented with Red or Black Pepper Oils

**DOI:** 10.3390/ani8100168

**Published:** 2018-10-02

**Authors:** Sameh Abdelnour, Mahmoud Alagawany, Mohamed E. Abd El-Hack, Asmaa M. Sheiha, Islam M. Saadeldin, Ayman A. Swelum

**Affiliations:** 1 Animal Production Department, Faculty of Agriculture, Zagazig University, Zagazig 44511, Egypt; samehtimor86@gmail.com (S.A.); asmaasheiha76@gmail.com (A.M.S.); 2 Poultry Department, Faculty of Agriculture, Zagazig University, Zagazig 44511, Egypt; dr.mahmoud.alagwany@gmail.com (M.A.); dr.mohamed.e.abdalhaq@gmail.com (M.E.A.E.-H.); 3 Department of Animal Production, College of Food and Agriculture Sciences, King Saud University, P.O. Box 2460, Riyadh 11451, Saudi Arabia; isaadeldin@ksu.edu.sa; 4 Department of Physiology, Faculty of Veterinary Medicine, Zagazig University, Zagazig 44511, Egypt; 5 Department of Theriogenology, Faculty of Veterinary Medicine, Zagazig University, Zagazig 44511, Egypt

**Keywords:** growing rabbits, red pepper, black pepper, growth, antioxidant indices, serum metabolites

## Abstract

**Simple Summary:**

After the ban on antibiotics as growth promoters in poultry and animal diets, nutritionists had to find safe and efficient alternatives. Because of their beneficial properties, the oils of red and black pepper were chosen to be supplemented to rabbit diets. Promising results were obtained with regard to the ability of these oils to improve rabbits’ growth performance, immunity, and antioxidant status.

**Abstract:**

The present study aimed to examine the impacts of the supplementation of red or black pepper oils to rabbit diet as growth promoters on New Zealand white (NZW) rabbits. One hundred and forty weaned NZW rabbits were divided randomly into seven groups in a completely randomized experiment using different quantities of red pepper oil (RPO; 0.5, 1.0, 1.5 g/kg diet) or black pepper oil (BPO; 0.5, 1.0, 1.5 g/kg diet), in addition to the control group. Compared to the control, values of live body weight (LBW) for rabbits fed either RPO or BPO enriched diets were greater. The concentrations of serum triglycerides and cholesterol were lower (*p* < 0.01) in the RPO- and BPO-treated groups than in the control. Immunity parameters and antioxidant indices were improved in treated groups in comparison to the control. Dietary RPO or BPO can affect some growth traits, improve immunity parameters and the antioxidant activity, and decrease the lipid profile and lipid peroxidation. The use of 0.5 g RPO/kg diet as a dietary supplement had a larger effect on growth parameters than the other treatment groups.

## 1. Introduction

Using different sources of growth promoters as dietary supplements to rabbit feed is a worldwide strategy to enhance the utilization of nutrients [1,2,3,4,5]. Plant-derived supplements are used to maintain the growth performance of livestock animals [6]. The active molecules in herbs or their oils can stimulate feed intake (FI), improve digestive enzyme secretion, and activate immune function, as well as promoting anthelmintic, antibacterial, antioxidant, and antiviral activities [7]. After banning antibiotics as growth enhancers, scientists researched natural and safe alternatives. Recently, plant-derived growth enhancers are used globally, as reported by Ortserga et al. [8]. 

Red pepper or *Capsicum annum* is used widely as a condiment and shows an assortment of pharmacological and physiological features [9,10]. In Latin and Central America, Asia, and Africa, it is common for red pepper to be used to increase the spice level of food [11]; however, it is well-known to be troublesome if consumed in excess [12,13]. Red pepper has many pharmacological and chemical properties like that of drugs’ classes which are capable of tissue deterioration inducing [14]. Over the past few decades, it was experimentally confirmed that a number of the common spices can show beneficial physiological activities [5]. The active compounds found in spices possess important roles in the promotion of physiological effects, including anti-oxidant and hypolipidemic activities [7,8,15].

Capsaicin (8-methyl-*N*-vanillyl-6-nonenamide), the active compound of pepper responsible for its spicy features, has captivated the interest of researchers for more than a century, who have proposed that it may have pharmacological and physiological effects [14]. Red pepper oil (RPO) showed many properties like antimicrobial, anti-oxidant, antitumor, antifungal, and anti-inflammatory activities [16,17].

Black pepper (*Piper nigrum*) is ordinarily consumed as a flavoring or used as a constituent in folk medicine. Remarkable uses for its active compound, termed ‘piperine,’ have been reported; for instance, it is used as a natural feed additive in livestock farming. Tatli et al. [18] postulated that of all-natural products used in this regard, piperine can most easily be made in bulk at low cost. Interestingly, it was identified that piperine has massive advantages over other natural products: it produces no residues in the animal meat, increases cell recovery, and promotes anti-apoptotic and anti-oxidative activities. This suggests that piperine could be beneficial when used in the event of a compromised immune system, which is usually treated with antibiotics [19,20].

Piperine induces adipocytes to produce energy from lipids and hastens body energy expenditure [21], as well as raising beta-endorphin production and serotonin level in the brain. Moreover, piperine can assuage gastrointestinal disturbances, cancer, and bronchitis by influencing gram positive bacteria and increasing the flow of digestive secretions in the stomach. However, there is inconsistent evidence about its effects on cancer [22]. Some previous studies investigated impacts of black or red pepper powder as a dietary supplement for poultry, but studies on the use of their oils are very scarce. So, the present study aimed to evaluate the influences of using red or black pepper oil as a natural additive on the growth performance, carcasses, blood constituents, oxidative status and serum metabolites in rabbits.

## 2. Materials and Methods 

The current experiment was carried out at the Rabbit Research Unit, Faculty of Agriculture, Zagazig University, Egypt. All the experimental procedures were carried out according to the Local Experimental Animal Care Committee and the ethics were approved by the institutional committee. Animals were cared for using husbandry guidelines derived from Zagazig University standard operating procedures.

### 2.1. Animals, Experimental Design, and Diets

A total number of 140 male New Zealand white growing weaned rabbits, at five weeks of the age with initial body weight of 635.71 ± 2.84 g were purchased from the Laboratory Animal Farm at Zagazig University. Rabbits were randomly divided into seven groups (7 groups × 5 rabbits × 4 rooms). The study continued for eight weeks and ended at 13 weeks of age. The dietary treatments used were as follows: C: control; RPO0.5: basal diet + 0.5 g RPO/kg diet; RPO1.0: basal diet + 1.0 g RPO/kg diet; RPO1.5: basal diet + 1.5 g RPO/kg diet; BPO0.5: basal diet + 0.5 g BPO/kg diet; BPO1.0: basal diet + 1.0 g BPO/kg diet; and BPO1.5: basal diet + 1.5 g BPO/kg diet.

Animals were kept in wire single cages (50 cm length × 30 cm width × 40 cm high). Feed and drinking water were offered on *ad libitum* basis. The animals’ health status was monitored throughout the study. All rabbits were reared under the same environmental, hygienic, and managerial conditions, and they were fed according to the nutritional requirements of the National Research Council (NRC). The RPO and BPO were bought from the Free Trade Egypt Company (Behira, Egypt). A cold pressing method was used to obtain oils from red pepper fruit, and black pepper seeds. The basal diet is shown in Table 1.

### 2.2. The Growth Performance Traits 

The feed intake (FI) and live body weight (LBW) were measured in replicates at biweekly intervals, and the body weight gain (BWG) and feed conversion ratio (FCR) were determined cumulatively through collected data by period.

### 2.3. The Carcass Traits

At the end of the study, six animals per group were taken, weighed, and exsanguinated. The carcasses were then prepared for analysis through removing skin, paws, feet, urinary bladder, genital organs, as well as alimentary tract. The hot carcass weights (main body, head, liver, lungs, heart, kidneys, and other the total edible parts) were measured [23]. The carcass parts were weighed, and the weights of the heart, spleen, legs, kidneys, skin, lungs and liver were registered and stated as g/kg of pre-slaughter weight. Carcass percentage = carcass weight × 100/LBW. Dressing percentage = (the carcass weight + the giblets weight) × 100/LBW.

### 2.4. Blood Hematology and Serum Metabolites 

The blood samples were harvested from the slaughtered rabbits and placed in sterile tubes. The hematological parameters were determined according to Schalm [24]. To determine serum metabolites, blood samples were left to clot and centrifuged at 3500 rpm for 15 min, after which the serum was isolated and stored at −20 °C until analysis. Serum metabolites were estimated using biodiagnostic kits and a spectrophotometer (Shimadzu, Kyoto, Japan) according to Akiba et al. [25]. For the antioxidant assay, the liver samples from six rabbits per treatment were homogenized (10% *weight*/*volume*) in potassium phosphate buffer solution (pH 7.4) and then centrifuged at 3000 rpm for 15 min. The obtained supernatants were then subjected to the measurements of glutathione peroxidase (before GSH-Px), catalase (CAT) and superoxide dismutase (SOD) activities, as well as malondialdehyde (MDA) and reduced glutathione (GSH) levels by the use of commercial kits bought from a spectrophotometer (Shimadzu, Kyoto, Japan) and Biodiagnostic Company (29 El-Tahrir St. Dokki, Giza, Egypt).

### 2.5. Statistics

All statistical analyses were performed using SAS (Statistical Analysis System) [26]. The data were assessed with a one-way ANOVA (with the diet as the fixed factor) using the post-hoc Newman Keuls test. The model utilized was as follows:

Y_ijk_ = μ + T_i_ + e_ij_, (1)

where Y_ijk_ = an observation, μ = the overall mean, T_i_ = effect of dietary treatments, and e_ij_ = random error. The significance was established at *p* < 0.05.

## 3. Results

### 3.1. Growth Performance 

**Figure 1:**
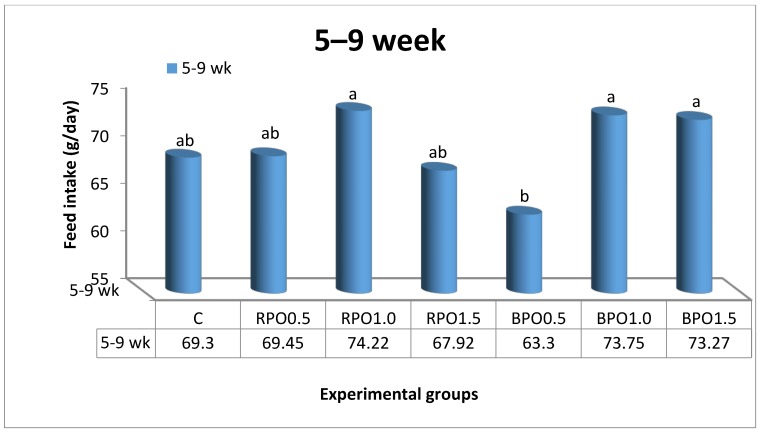
Feed intake of growing NZW rabbits as affected by dietary treatments during 5–9 weeks of age. (C: control; RPO0.5 (0.5 g red pepper oil): basal diet + 0.5 g RPO/kg diet; RPO1.0: basal diet + 1.0 g RPO/kg diet; RPO1.5: basal diet + 1.5 g RPO/kg diet; BPO0.5 (0.5 g black pepper oil): basal diet + 0.5 g BPO/kg diet; BPO1.0: basal diet + 1.0 g BPO/kg diet; and BPO1.5: basal diet + 1.5 g BPO/kg diet). a, b: superscript letters showing significantly difference (*p* < 0.05).

**Figure 2:**
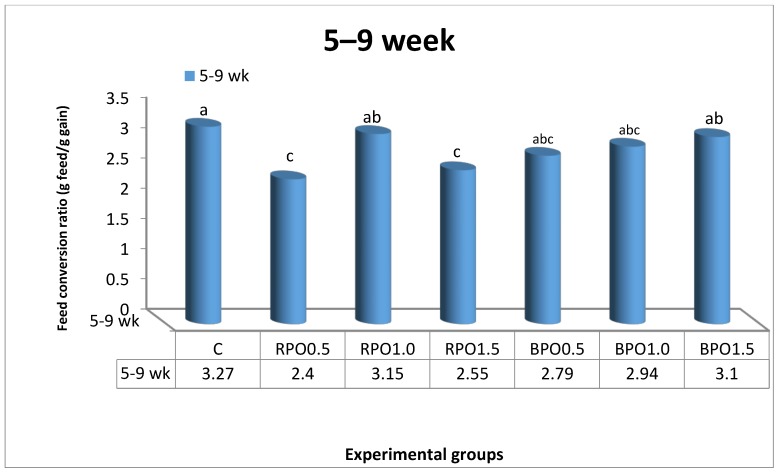
Feed conversion ratio of growing NZW rabbits as affected by dietary treatments during 5–9 weeks of age. (C: control; RPO0.5 (0.5 g red pepper oil): basal diet + 0.5 g RPO/kg diet; RPO1.0: basal diet + 1.0 g RPO/kg diet; RPO1.5: basal diet + 1.5 g RPO/kg diet; BPO0.5 (0.5 g black pepper oil): basal diet + 0.5 g BPO/kg diet; BPO1.0: basal diet + 1.0 g BPO/kg diet; and BPO1.5: basal diet + 1.5 g BPO/kg diet). a, b: superscript letters showing significantly difference (*p* < 0.05).

The effects of dietary RPO and BPO supplements on the growth indices of growing NZW rabbits throughout the experiment are illustrated in Table 2 and Figure 1 and Figure 2. Generally, values of LBW for rabbits fed either RPO- or BPO-enriched diets were higher (*p* < 0.05) than those in the control group at nine weeks of age (Table 2). However, RPO supplementation (0.5 and 1.5 g/kg diet) was superior to BPO in its effect on LBW. It is worth noting that the highest (*p* < 0.05) LBW (1481.67 g) and BWG (29.17 g) values were recorded in the group that received 0.5 g/kg RPO, compared to other treatment groups. In comparison to the control, supplementing the diet with BPO increased (*p* < 0.05) the LBW by about 1.87%, 2.96%, and 1.26% for the levels of 0.5, 1.0, and 1.5 BPO/kg diet, respectively, at nine weeks of age (Table 2). Figure 1 shows a significant (*p* < 0.05) effect of dietary treatments on FI only during the first period (5–9 weeks of age). Rabbits that received RPO1.0, BPO1.0, and BPO1.5 diets consumed more feed than others. In the present study, during the period of five to nine weeks of age, the best FCR was detected in rabbits fed the RPO0.5 diet compared to those in other treatment groups (Figure 2). 

As shown in Figure 2, FCR improved by 14.68%, 10.09% and 5.20% in the groups that received 0.5, 1.0, and 1.5 BPO/kg diet, respectively, within the period of five to nine weeks of age. 

### 3.2. Carcass Traits

The data in Table 3 highlight the effect of dietary treatments on the carcass and edible organ relative weights, as well as the cecum length. Only the relative weights of liver and spleen were statistically (*p* < 0.05) different as a result of the dietary treatments. The heaviest liver and spleen were found in the RPO0.5 group compared to those in the other experimental groups.

### 3.3. Blood Hematology

Data in Table 4 illustrate the effects of dietary RPO and BPO supplementation on hematological parameters. No significant differences were noticed in any of hematological traits except for hemoglobin content, mean corpuscular volume, and platelet count. No significant differences were observed in HGB between all treatment groups and control. However, the lowest value of HGB was recorded in RPO0.5 group in comparison with BPO groups. Blood content of MCV was not significantly (*p* < 0.05) different in animals given BPO diets compared to those in the control group. On the other hand, RPO supplementation (RPO1.0 and RPO1.5) decreased (*p* < 0.01) the blood content of MCV compared to those in the control. In comparison with control, red pepper at all levels and black at 0.5 g/kg did not significantly influence the content of PLT (platelets). In a converse trend, highly significant (*p* < 0.01) increases in the aforementioned parameter were detected in the experimental groups that received BPO (BPO1.0 and BPO1.5) compared to those in the control and RPO groups. 

### 3.4. Serum Biochemistry 

As shown in Table 5, the majority of blood metabolites were statistically (*p* < 0.05 or 0.01) different in rabbits given dietary treatments compared to those in the control group. For liver function, ALT activity was depressed (*p* < 0.01) due to dietary treatments when compared to control group. In apart from RPO1.0 and BPO0.5 groups, activity of AST (aspartate amino transferase) was not significantly (*p* = 0.004) different in animals given BPO or RPO diets except compared to those in the control group. The highest value of AST was recorded in RPO1.0 group, while the lowest one was recorded in BPO0.5 group. 

For kidney function, supplementing rabbit diet with either RPO or BPO (except for the RPO0.5 and BPO1.0 groups) decreased (*p* < 0.01) the serum creatinine content. In comparison with control, a significant increase (*p* < 0.05) was recorded in the serum concentration of total protein (TP) as a result of enriching rabbit diet with RPO and BPO. The serum concentration of albumin was increased as a result of enriching rabbit diet with RPO, compared to those in the control group. A significant increase (*p* < 0.05) was recorded in the serum concentration of albumin as a result of enriching rabbit diet with RPO, compared to those in the control group. But, dietary supplementation of BPO did not affect the serum concentration of albumin when compared to control. In apart from control, no significant differences were observed in albumin among treatment groups. 

The concentrations of serum total cholesterol (TC) and low-density lipoprotein (LDL) were decreased (*p* < 0.05) in RPO- and BPO-treated groups in comparison to the control. Dietary supplementation of RPO1.0 was superior to all treatments in its effect on high-density lipoprotein (HDL). In comparison with the control and BPO1.5 groups, a significant decrease (*p* < 0.01) was recorded in the serum concentration of triglyceride (TG) as a result of enriching rabbit diet with RPO and BPO. 

### 3.5. Antioxidant and Immunity Indices 

In the current study, a significantly positive impact of phytogenic additives was seen on immunological patterns like immunoglobulin G (IgG), which was significantly (*p* < 0.01) enhanced in groups (BPO0.5, RPO1.0, and RPO1.5) compared to that of the control group (Table 6). 

The effects of dietary RPO and BPO supplements on anti-oxidant activities concerning the total antioxidant capacity (TAC), and MDA, GSH and SOD content of growing rabbits are clarified in Table 6. All the aforementioned patterns were statistically (*p* < 0.01) affected by the dietary treatments. As compared to control, the levels of TAC were significantly (*p* = 0.002) increased only in the RPO0.05 and RPO1.0 groups. The activity of SOD was significantly (*p* < 0.01) improved in the RPO-treated groups. But, BPO supplements at levels of 0.5 and 1 g/kg diet did not affect SOD when compared to control.

Enriching rabbit diets with 0.5 g RPO/kg diet produced the best activities of TAC and SOD compared to those in other groups. Conversely, the highest (*p* = 0.005) concentrations of MDA and GSH were recorded in the control group. Supplementing rabbit diets with either RPO or BPO depressed levels of MDA and GSH compared to those in the control.

## 4. Discussion 

The analysis of RPO and BPO has been reported [5,6]. The Brazilian red pepper essential oil (RPEO) has abundant properties of interest, including antimicrobial, anti-inflammatory and antioxidant and activities, it can be used as a feed additive for weanling pigs [8]. BPO is a powerful natural antioxidant that can slow down the oxidation of fats [25]. Furthermore, BPO has a noteworthy antibacterial activity against both *Staphylococcus aureus* and *Escherichia coli*. BPO displayed significant anti-proliferative activity in dermal fibroblast cells. As well, BPO significantly inhibited the production of Collagen I and III as well as plasminogen activator inhibitor 1. At the gene level, BPO robustly modulated various genes and signaling pathways critical for tissue remodeling, metabolism, and cancer biology [26]. 

Values of LBW for rabbits fed either RPO- or BPO- enriched diets were higher (*p* < 0.05) than those in the control group at nine weeks of age (Table 2). The improvement in LBW and BWG by RPO or BPO as phytogenic additives may be due to the presence of some constituents that promote the digestion and absorption of nutrients. Additionally, Alagawany et al. [27] suggested that it might be the biological activity of the components of RPO that enhance the FI and promote the growth rate. In fact, numerous in vitro and in vivo studies that have used active components (e.g., tannins, saponins, and flavonoids) extracted from herb extracts, have also reported antifungal, antimicrobial, anti-inflammatory, and anti-oxidant activities [28]. Silva et al. [29] confirmed that the supplementation of 4.0 g/kg RPO to broiler diets increased LBW and BWG compared to those did not receive antimicrobials and indicating that higher doses of RPO might be needed as a feed additive to achieve growth enhancing properties. Conversely, Cairo et al. [30] stated that RPO did not impact growth of animals.

Results showed that supplementing the diet with BPO increased (*p* < 0.05) the LBW compared to control (Table 2). In parallel, Puvača et al. [31] postulated that adding black pepper to broiler diets has a positive impact on growth rate of chickens; this is in accordance with the antecedent studies of Al-Kassie et al. [22] and Valiollahi et al. [32]. Additionally, Abou-Elkhair et al. [33] showed that the addition of black pepper, or a mixture of turmeric powder and black pepper, to broiler chicken diets led to a higher final body weight of chickens during the fattening period of 35 days. The improvement in broiler body weight as a result of supplementation with black pepper powder was also observed and reported by Ghazalah et al. [34]. Hosseini et al. [35] observed that black pepper supplements can improve digestion and absorption of nutrients through increasing the secretion of digestive enzymes in the stomach and destroying infectious bacteria. The influence of black pepper is related to higher absorption, which augments the secretion of digestive enzymes and also decreases the velocity of material transit. The most active component in black pepper, piperine, promotes pancreatic digestive enzymes such as amylase, lipase, and protease, which play critical roles in digestion process [36]. 

In accordance with our findings, Al-Kassie et al. [22] demonstrated that the active compound capsaicin, which is rich in vitamin C, improves feed consumption and positively influences the body weight value. Moreover, Ghazalah et al. [34] and Tollba et al. [37] indicated that using a moderate level of black pepper stimulates feed consumption and causes the high piperazine citrate activity, which alters the flow of digestive fluids in the stomach. It has been reported that some botanicals such as RPO have the capacity to stimulate the endogenous production of enzymes, bile acids, or pancreatic juices, which improve nutrient digestibility, and thus, positively affect the FCR. In a previous study, Ipharraguerre et al. [38] observed that red pepper supplementation improved dietary fat digestibility when supplemental fat was included at 4%. The findings of our study are similar to those of Al-Harthi [39], who revealed that the supplementation of hot red pepper to chick diet improved FCR and induced digestion, because of its carminative property and antimicrobial attributes. The improvement in FCR in the present study may be due to the ability of black pepper to improve the digestibility of the feed which would improve the FCR and other growth parameters [40]. However, this improvement was insignificant according to Al-Harthi [39]. Conversely, Al-Kassie et al. [22] and Abou-Elkhair et al. [33] both reported that the use of black pepper powder in chicken feed did not have a positive influence on the FCR.

Results in Table 3 showed that the majority of the carcass and edible organ percentages were not significantly affected by dietary treatments. In partial agreement with our findings, Cairo et al. [30] observed no differences in the relative size of edible organs between animals fed diets supplemented with different quantities of RPO. Additionally, Costa et al. [41], reported no differences in the organ weights of animals fed diets supplemented with essential oils of thyme, cinnamon, eucalyptus, *Melaleuca alternifolia*, *Echinacea angustifolia,* ginger and pepper extracts. Al-Kassie et al. [22] stated that a mixture of red and black pepper powder did not exert any significant effects on the carcass or organ weights of broilers. In contrast with our results, Rahimian et al. [42] postulated that liver relative weight was significantly increased (*p* < 0.05) in broilers fed with black pepper compared to those in the control.

The supplementation with RPO decreased (*p* < 0.01) the blood content of hemoglobin, mean corpuscular volume, and platelet count compared to those in the control (Table 4). Al-Kassie et al. [22] showed that broilers fed with a black and red pepper mixture had a significantly lower red blood cell count, packed cell volume, and hemoglobin level compared to those in the control group.

Our results showed better liver and kidney function in rabbits fed RPO or BPO than the control (Table 5). Corduk et al. [43] stated that the rapid metabolization of essential oils in the liver can damage it and consequently increase serum content of the liver enzymes (AST and ALT). However, our findings related to the serum AST and ALT levels showed that the levels of RPO and BPO were safe and useful in expressing renal and liver function. Similarly, Traesel et al. [44] postulated that the prolonged use of high levels of supplementary essential oils could not cause nephritis or renal failure. 

A significant increase (*p* < 0.05) was reported in the serum TP and albumin as a response to RPO and BPO supplementation (Table 5). This increase in serum TP and albumin may be due to the bioactive components in RPO and BPO. On the contrary, Dabbou et al. [7] and Kovitvadhi et al. [45] mentioned that dietary phytogenic supplementation did not affect the blood parameters (TP, globulin, and albumin) or humoral immune responses in growing rabbits.

Levels of serum TC, triglyceride, LDL were decreased (*p* < 0.01) and serum HDL was increased (*p* < 0.01) in RPO- and BPO-treated groups compared to those in the control. The reduction in blood cholesterol may be due to the decrease in the activity of enzyme synthesis, as hypothesized by Chowdhury et al. [46]. On the other hand, Srinivasan and Satyanarayana [47] indicated that capsaicin is considered the active component of red hot peppers and feeding female rats with red hot pepper depressed their serum triglyceride contents. In accordance with our results, Puvača et al. [31] found that supplementation with 1.0 g/100 g of black pepper significantly (*p* < 0.05) decreased the levels of triglycerides in the blood serum in broilers. The authors added that this result can be clarified by the conceivable decrease in acetyl-CoA enzyme synthesis that is important for the biosynthesis of fatty acids. In the present study, the addition of either RPO or BPO to rabbit diet decreased the serum LDL and increased the serum HDL. This influence could be elucidated by the possible mechanism of anti-oxidant and antiperoxide activity decreasing LDL, or the decline in hepatic production of very low-density lipoprotein (VLDL) which is a precursor of LDL in the bloodstream [48]. Ghaedi et al. [49] found that the addition of black pepper to the diet of broilers decreased triglycerides and total cholesterol, while the concentration of HDL increased. Moreover, Al-Kassie et al. [22] reported that broilers fed with black pepper and red pepper mixture had significantly lowered cholesterol. Authors theorized that the addition of spices or herbs to animal feed can facilitate the activity of enzymes that participate in the transformation of cholesterol to bilious acids, and subsequently will result in a lower cholesterol concentration in the carcass. In disagreement with our results, Corduk et al. [43] found no significant influences on the serum total protein, triglyceride, cholesterol, creatinine, and alanine aminotransferase enzyme contents after RPO addition to broiler diets. Similarly, Al-Harthi [39] described that a combination of cumin, cardamom, and black and red pepper at 2 and 4 g/kg did not significantly influence the TC, TP, or ALT enzyme contents.

The enhancement of immune functions in treated groups (Table 6) may imply herbal supplements are rich in flavonoids, which act as strong anti-oxidants [50]. The supplementation of RPO and BPO may enhance the immune function via augmentation of the immunoglobulin (IgM) levels in rabbits fed phytogenic feed additives compared to those in the control group. The positive effects of RPO and BPO might be due to their antibacterial, antioxidant, and anti-inflammatory properties. These phytogenic additives are proposed to lower the growth and colonization of pathogenic and non-pathogenic species of bacteria in the gut of rabbits, and to balance microbial ecosystems in the gut, which all contribute to better feed utilization and metabolism [51]. These findings are similar to the results of Alagawany et al. [27], who reported that supplementation of 400 and 600 mg/kg diet of yucca powder showed greater levels of IgM and IgG in rabbit blood compared to that in the control group. 

In the RPO- and BPO-treated groups, the levels of TAC and SOD were significantly (*p* < 0.01) increased; while the highest (*p* = 0.005) concentration of MDA was recorded in the control group. The SOD plays a major role in protecting cells from oxidative damage; this process requires specific nutrients to be present in the diet [6]. The present conclusions are in accordance with those of Lin et al. [52], who reported that herb intake caused an increase in serum antioxidant enzyme activities and a decrease in MDA levels. Generally, it was reported that essential oils of pepper enhance amino acid absorption, such as methionine or cysteine, in the gastro-intestinal tract which are the limiting factors of glutathione synthesis [22,30,42]. The reduction in glutathione in treated groups might be due to the inhibition the formation of glutathione disulfide; thereby glutathione can protect cells from the attack of free radicals, prevent from the oxidative damage of macromolecules, and inhibit apolipoprotein B protein peroxidation [27]. Alagawany et al. [27] showed that dietary supplementation of herbs to rabbit diets had positive effects on both SOD and TAC activities. From these findings, it could be proposed that supplements with natural antioxidants could be practical in the future to enhance the health status of rabbits.

## 5. Conclusions

From our results, it can be concluded that dietary RPO or BPO supplementation significantly increased growth in rabbits and improved the immunity parameters. Additionally, rabbits fed a diet enriched with RPO or BPO showed decreased lipid profile and lipid peroxidation, and an improved anti-oxidant activity. The use of RPO was more effective than BPO in terms of its effect on growth performance traits, particularly at the level of 0.5 g RPO/kg diet.

## Figures and Tables

**Table 1:** Ingredients and composition of basal diet of growing rabbits (as fed).

Items	Basal Diet
Ingredient	%
Maize	20
Soybean meal	20
Wheat bran	16
Berseem hay	30
Barley grain	10
Molasses	2
Limestone	1
NaCl	0.5
Premix *	0.5
Calculated composition, %	
ME, MJ/kg	7.95
Crude protein	17.50
Calcium	0.88
Available phosphorus	0.20
Analyzed composition (%, on DM basis)	
Crude protein	16.54
Ether extract	2.25
Crude fiber	12.33
Dry matter	88.06
Organic matter	90.57
Ash	9.43
Nitrogen-free extract	59.45

* Each 1 kg of premix (minerals and vitamins mixture) contains vit. A, 20,000 IU; vit. D3, 15,000 IU; vit. E, 8.33 g; vit. K, 0.33 g; vit. B1, 0.33 g; vit. B2, 1.0 g; vit. B6, 0.33 g; vit. B5, 8.33 g; vit. B12, 1.7 mg; pantothenic acid, 3.33 g; biotin, 33 mg; folic acid, 0.83 g; choline chloride, 200 g.

**Table 2:** Live body weight and body weight gain of growing New Zealand white (NZW) rabbits as affected by dietary treatments at 13 weeks of age.

Items	Live Body Weight (g)			Body Weight Gain (g)		
	5 week	9 week	13 week	5–9 week	9–13 week	5–13 week
C	711.67	1310.00 ^c^	2063.33	21.37 ^c^	26.90	24.14
RPO0.5	665.00	1481.67 ^a^	1995.00	29.17 ^a^	18.33	23.75
RPO1.0	676.67	1345.00 ^b,c^	1948.33	23.87 ^b,c^	21.55	22.71
RPO1.5	668.33	1423.33 ^b^	2086.67	26.97 ^b^	23.69	25.33
BPO0.5	697.50	1335.00 ^b,c^	2125.00	22.77 ^b,c^	28.21	25.49
BPO1.0	646.67	1350.00 ^b,c^	2165.00	25.12 ^a,b,c^	29.11	27.11
BPO1.5	661.67	1326.67 ^b,c^	1931.67	23.75 ^b,c^	21.61	22.68
SEM	8.98	16.33	35.87	0.72	1.31	0.63
*p*-value	0.562	0.020	0.561	0.039	0.233	0.511

C: control; RPO0.5 (0.5 g red pepper oil): basal diet + 0.5 g RPO/kg diet; RPO1.0: basal diet + 1.0 g RPO/kg diet; RPO1.5: basal diet + 1.5 g RPO/kg diet; BPO0.5 (0.5 g black pepper oil): basal diet + 0.5 g BPO/kg diet; BPO1.0: basal diet + 1.0 g BPO/kg diet; BPO1.5: basal diet + 1.5 g BPO/kg diet. RPO: Red pepper oil; BPO: Black pepper oil. SEM: Standard error of mean. Means in the same column with no superscript letters after them or with a common superscript letter following them are not significantly different (*p* < 0.05).

**Table 3:** Carcass traits of growing NZW rabbits as affected by dietary treatments at 13 weeks of age.

Items	Carcass Traits (as % of Pre-Slaughter Weight)							Cecum Length (cm)
	Carcass	Dressing	Liver	Heart	Lung	Kidney	Spleen	
C	54.92	45.08	4.43 ^a,b^	0.29	0.68	0.99	0.08 ^a,b^	11.60
RPO0.5	54.78	45.22	5.38 ^a^	0.28	0.63	0.86	0.09 ^a^	12.23
RPO1.0	57.84	42.16	4.15 ^b^	0.27	0.79	0.85	0.06 ^b^	12.27
RPO1.5	52.85	47.15	4.44 ^a,b^	0.27	0.74	0.76	0.06 ^b^	11.80
BPO0.5	52.94	47.06	3.88 ^b^	0.30	0.78	0.94	0.08 ^a,b^	11.50
BPO1.0	56.08	43.92	3.41 ^b^	0.35	0.71	0.89	0.07 ^a,b^	11.53
BPO1.5	57.37	42.63	3.87 ^b^	0.29	0.68	0.81	0.08 ^a,b^	11.73
SEM	1.02	1.02	0.17	0.01	0.03	0.03	0.003	0.24
*p*-value	0.840	0.840	0.042	0.711	0.726	0.280	0.041	0.971

C: control; RPO0.5: basal diet + 0.5 g RPO/kg diet; RPO1.0: basal diet + 1.0 g RPO/kg diet; RPO1.5: basal diet + 1.5 g RPO/kg diet; BPO0.5: basal diet + 0.5 g BPO/kg diet; BPO1.0: basal diet + 1.0 g BPO/kg diet; and BPO1.5: basal diet + 1.5 g BPO/kg diet. RPO: Red pepper oil; BPO: Black pepper oil. SEM: Standard error of mean. Means in the same column with no superscript letters after them or with a common superscript letter following them are not significantly different (*p* < 0.05).

**Table 4:** Blood hematology of growing NZW rabbits as affected by dietary treatments at 13 weeks of age.

Items	WBCs (10^3^/μL)	LYM (%)	MID (%)	GRA (%)	RBCs (10^6^/μL)	HGB (g/dL)	HCT (%)	MCV (µm^3^)	MCH (pg)	PLT (10^3^/μL)
C	5.71	52.60	6.50	40.90	4.18	11.20 ^a,b,c^	32.30	77.40 ^a^	26.85	296.50 ^b,c^
RPO0.5	8.57	54.97	6.07	38.97	4.13	9.83 ^c^	30.20	73.30 ^a,b^	26.57	173.33 ^c^
RPO1.0	6.20	43.10	7.80	49.10	4.69	10.60 ^b,c^	32.70	69.90 ^b^	22.60	190.50 ^c^
RPO1.5	4.83	53.13	6.27	40.60	4.74	10.83 ^b,c^	33.33	70.33 ^b^	22.80	167.67 ^c^
BPO0.5	6.35	44.80	5.37	40.80	4.83	12.41 ^a^	36.33	75.33 ^a^	25.73	364.67 ^a,b^
BPO1.0	7.42	52.95	6.30	40.55	5.13	12.55 ^a^	39.90	77.80 ^a^	25.00	478.50 ^a^
BPO1.5	7.11	48.73	6.77	44.40	4.54	12.13 ^a,b^	34.63	76.17 ^a^	26.77	478.33 ^a^
SEM	0.40	1.53	0.25	1.31	0.11	0.26	0.91	0.81	0.55	31.09
*p*-value	0.253	0.261	0.306	0.464	0.183	0.008	0.079	0.006	0.130	<0.001

C: control; RPO0.5: basal diet + 0.5 g RPO/kg diet; RPO1.0: basal diet + 1.0 g RPO/kg diet; RPO1.5: basal diet + 1.5 g RPO/kg diet; BPO0.5: basal diet + 0.5 g BPO/kg diet; BPO1.0: basal diet + 1.0 g BPO/kg diet; and BPO1.5: basal diet + 1.5 g BPO/kg diet. RPO: Red pepper oil; BPO: Black pepper oil. WBCs: white blood cells; LYM: lymphocytes; MID: mid-range; GRA: granulocytes; RBCs: red blood cells; HGB: hemoglobin; HCT: hematocrit; MCV: Mean corpuscular volume; MCH: Mean corpuscular hemoglobin; PLT: Platelet count. SEM: Standard error of mean. Means in the same column with no superscript letters after them or with a common superscript letter following them are not significantly different (*p* < 0.05).

**Table 5:** Blood profiles of growing NZW rabbits as affected by dietary treatments at 13 weeks of age.

Items	ALT (IU/L)	AST (IU/L)	TP (g/dL)	Alb (g/dL)	Glob (g/dL)	A/G (%)	Creatinine (mg/dL)	TC (mg/dL)	TG (mg/dL)	HDL (mg/dL)	LDL (mg/dL)
C	82.22 ^a^	32.98 ^b,c^	5.67 ^b^	3.16 ^b^	2.51	1.26	0.89 ^a^	215.99 ^a^	76.06 ^a^	39.62 ^b^	36.06 ^a^
RPO0.5	67.97 ^b,c^	30.98 ^b,c,d^	7.10 ^a^	4.24 ^a^	2.86	1.48	0.86 ^a,b^	198.46 ^b^	58.20 ^b^	39.23 ^b^	21.88 ^c^
RPO1.0	68.76 ^b,c^	26.11 ^d^	6.73 ^a^	4.01 ^a^	2.72	1.48	0.83 ^b^	187.80 ^b,c^	51.13 ^b^	48.34 ^a^	24.44 ^b,c^
RPO1.5	73.00 ^b^	35.86 ^a,b^	6.85 ^a^	3.95 ^a^	2.90	1.36	0.78 ^c^	181.35 ^c^	57.21 ^b^	39.32 ^b^	26.39 ^b^
BPO0.5	60.33 ^c^	39.47 ^a^	6.64 ^a^	3.74 ^a,b^	2.90	1.29	0.82 ^b^	175.42 ^c^	60.24 ^b^	33.37 ^b^	22.76 ^b,c^
BPO1.0	74.99 ^b^	29.74 ^c,d^	6.54 ^a^	3.68 ^a,b^	2.86	1.29	0.87 ^a,b^	188.58 ^b,c^	58.05 ^b^	35.26 ^b^	22.26 ^c^
BPO1.5	64.37 ^b,c^	31.12 ^c^	6.50 ^a^	3.58 ^a,b^	2.92	1.24	0.77 ^c^	179.06 ^c^	72.83 ^a^	41.26 ^a,b^	21.56 ^c^
SEM	2.11	1.06	0.12	0.42	0.04	0.03	0.02	3.20	2.13	1.24	1.13
*p*-value	< 0.001	0.004	0.014	0.036	0.072	0.241	0.003	<0.001	0.001	0.013	<0.001

C: control; RPO0.5: basal diet + 0.5 g RPO/kg diet; RPO1.0: basal diet + 1.0 g RPO/kg diet; RPO1.5: basal diet + 1.5 g RPO/kg diet; BPO0.5: basal diet + 0.5 g BPO/kg diet; BPO1.0: basal diet + 1.0 g BPO/kg diet; and BPO1.5: basal diet + 1.5 g BPO/kg diet. RPO: Red pepper oil; BPO: Black pepper oil. ALT: alanine aminotransferase; AST: aspartate aminotransferase; TP: total protein; Alb: albumin; Glob: globulin; A/G: albumin/ globulin ratio; TC: total cholesterol; TG: triglycerides; HDL: high density lipoprotein; LDL: low density lipoprotein. SEM: Standard error of mean. Means in the same column with no superscript letters after them or with a common superscript letter following them are not significantly different (*p* < 0.05).

**Table 6:** Immunity and oxidative status of growing NZW rabbits as affected by dietary treatments at 13 weeks of age.

Items	Immunity Parameters		Oxidative Status			
	IgG (mg/dL)	IgM (mg/dL)	TAC (ng/mL)	SOD (U/mL)	MDA (nmol/mL)	GSH (ng/mL)
C	48.29 ^c^	74.61	0.22 ^c,d^	0.17 ^d^	3.53 ^a^	0.44 ^a^
RPO0.5	50.47 ^b,c^	75.27	0.29 ^a^	0.28 ^a^	2.14 ^b^	0.22 ^b^
RPO1.0	52.51 ^a,b^	77.24	0.27 ^a,b^	0.27 ^a^	1.35 ^b^	0.24 ^b^
RPO1.5	53.70 ^a^	75.81	0.25 ^a,b,c^	0.23 ^b^	1.17 ^b^	0.21 ^b^
BPO0.5	52.50 ^a,b^	75.64	0.21 ^c,d^	0.16 ^d^	1.10 ^b^	0.13 ^b^
BPO1.0	50.76 ^b,c^	74.19	0.19 ^d^	0.18 ^c,d^	1.39 ^b^	0.17 ^b^
BPO1.5	49.12 ^c^	76.45	0.24 ^b,c^	0.21 ^b,c^	1.47 ^b^	0.16 ^b^
SEM	0.47	0.53	0.01	0.01	0.22	0.03
*p*-value	0.002	0.827	0.002	<0.001	0.005	0.008

C: control; RPO0.5: basal diet + 0.5 g RPO/kg diet; RPO1.0: basal diet + 1.0 g RPO/kg diet; RPO1.5: basal diet + 1.5 g RPO/kg diet; BPO0.5: basal diet + 0.5 g BPO/kg diet; BPO1.0: basal diet + 1.0 g BPO/kg diet; BPO1.5: basal diet + 1.5 g BPO/kg diet. RPO: Red pepper oil; BPO: Black pepper oil. IgG: immunoglobulin G: IgM: immunoglobulin M; TAC: total antioxidant capacity; SOD: superoxide dismutase; MDA: malondialdehyde; GSH: glutathione. SEM: Standard error of mean. Means in the same column with no superscript letters after them or with a common superscript letter following them are not significantly different (*p* < 0.05).

## Abbreviations

NZW	New Zealand white rabbits;
FCR	feed conversion ratio;
LBW	live body weight;
FI	feed intake;
BWG	body weight gain;
RPO	Red pepper oil;
BPO	Black pepper oil;
SEM	Standard error of mean;
WBCs	white blood cells;
LYM	lymphocytes;
MID	mid-range;
GRA	granulocytes;
RBCs	red blood cells;
HGB	hemoglobin;
HCT	hematocrit;
MCV	Mean corpuscular volume;
MCH	Mean corpuscular hemoglobin;
PLT	Platelet count;
ALT	alanine aminotransferase;
AST	aspartate aminotransferase;
TP	total protein;
Alb	albumin;
Glob	globulin;
A/G	albumin/globulin ratio;
TC	total cholesterol;
TG	triglycerides;
HDL	high density lipoprotein;
LDL	low density lipoprotein;
IgG	immunoglobulin G;
IgM	immunoglobulin M;
TAC	total antioxidant capacity;
SOD	superoxide dismutase;
MDA	malondialdehyde;
GSH	glutathione.
